# Subcutaneous Injections of Turpentine in Pneumonia

**Published:** 1895-06

**Authors:** W. H. Benjamin


### SELECTIONS.

A CASE OF RELAPSE IN PNEUMONIA IN WHICH SUBCUTANEOUS INJECTIONS OF THE ESSENCE OF TURPENTINE APPEAR TO HAVE BEEN FOLLOWED BY GOOD RESULTS.—It is not my intention to go into the history of these injections which are practised by a certain number of veterinarians, and which Dr. Fochier, of Lyons, has recommended to give birth to abscesses which he names as abscesses of fixation. Professor Dieulafoy, in his manual of *Internal Pathology*, in the chapter on the treatment of pneumonia, relates two cases of cure which he has obtained in two women with very severe attacks of pneumonia, probably at the point of suppuration. He adds, which seems remarkable, that the pus from the focus was *amicrobien.* My desire is simply to publish a fact interesting enough to induce those who wish to follow my example.

On March 8, 1894, I was called to attend a gelding, of heavy draught, black, seven years old, but recently arrived from Paris. The animal, which that morning had refused his ration of oats, presented the symptoms of beginning infectious pneumonia. The temperature was elevated, 40.4°, and a localization of the disease seemed to have appeared on the left side. Under the influence of a very large sinapism to the chest there was on the next day and the day following an amelioration, at least a sort of momentary calm, such as one often enough observes in similar cases; the temperature was 39.8° to 39.5°. On the 11th the pathological picture was singularly modified: the temperature was 40.5°, and auscultation revealed the sign of double pneumonia. Four litres of blood were withdrawn and two vesicants applied to the chest. I pass over at this time the internal treatment, which was not unusual. Three days later the temperature fell to 38.5°, and, after oscillating for some days between 38.9° and 39.9°, fell on the 19th to 37.6°, and the animal appeared convalescent. He ate, lay down, and appeared to be recovering.

On the 26th he sustained a relapse. He was then in a very alarming state. Staggering gait, greatly injected conjunctivæ, extremely difficult respiration, flank movements, growing evidence of pleurisy complications, pulse quick and small, temperature 40.4°, appetite gone. Such was an exact picture of the symptoms which I observed. I then conceived the idea of making subcutaneous injections of the essence of turpentine. I obtained a Pravas syringe which contained exactly 3 grammes 50 centigrammes of this liquid. I made two injections, in all 7 grammes. There followed that evening a very voluminous œdema, and the next day toward the latter part of the afternoon the temperature began to fall and the general symptoms were less alarming. The œdema increased more and more, without, however, causing apparent suffering to the patient. A manifest fluctuation appeared, and on the 2d of April I punctured the abscess in two places, letting out about half a litre of white pus streaked with blood, thick, gluey, colloid, exhaling a strong odor of the essence of turpentine. The improvement continued, the alarming symptoms disappeared, the lungs slowly became permeable to air, and the animal entered this time definitely into a period of convalescence. The respirations remaining a little accelerated I submitted the animal to an arsenical treatment, in the course of which the discharge of mucus was quite abundant. The pockets of the abscesses cicatrized rapidly, and to-day it would be difficult to find a trace of their existence.—W. H. BENJAMIN, *in the Recueil de Médécine Vétérinaire*, April 30, 1895.

